# A New Hydroxychavicol Dimer from the Roots of *Piper betle*

**DOI:** 10.3390/molecules18032563

**Published:** 2013-02-26

**Authors:** Chwan-Fwu Lin, Tsong-Long Hwang, Chun-Chien Chien, Huei-Yu Tu, Horng-Liang Lay

**Affiliations:** 1 Department of Cosmetic Science, Chang Gung University of Science and Technology, Taoyuan 333, Taiwan; E-Mail: cflin@gw.cgust.edu.tw; 2 Research Center for Industry of Human Ecology, Chang Gung University of Science and Technology, Taoyuan 333, Taiwan; 3 Cell Pharmacology Laboratory, Graduate Institute of Natural Products, College of Medicine, Chang Gung University, Taoyuan 333, Taiwan; E-Mail: htl@mail.cgu.edu.tw; 4 Chinese Herbal Medicine Research Team, Healthy Aging Research Center, Chang Gung University, Taoyuan 333, Taiwan; 5 Department of Dentistry, Chang Gung Memorial Hospital, Taoyuan 333, Taiwan; E-Mail: meridian_chien@hotmail.com; 6 Department of Plant Industry, National Pingtung University of Science and Technolog, Pingtung 912, Taiwan; E-Mail: layhl@mail.npust.edu.tw

**Keywords:** *Piper betle*, 2-(γ'-hydroxychavicol)-hydroxychavicol, hydroxychavciol, superoxide anion, elastase, neutrophils

## Abstract

A new hydroxychavicol dimer, 2-(γ'-hydroxychavicol)-hydroxychavicol (**1**), was isolated from the roots of *Piper betle* Linn. along with five known compounds, hydroxychavicol (**2**), aristololactam A II (**3**), aristololactam B II (**4**), piperolactam A (**5**) and cepharadione A (**6**). The structures of these isolated compounds were elucidated by spectroscopic methods. Compounds **1** and **2** exhibited inhibitory effects on the generation of superoxide anion and the release of elastase by human neutrophils.

## 1. Introduction

**Figure 1:**
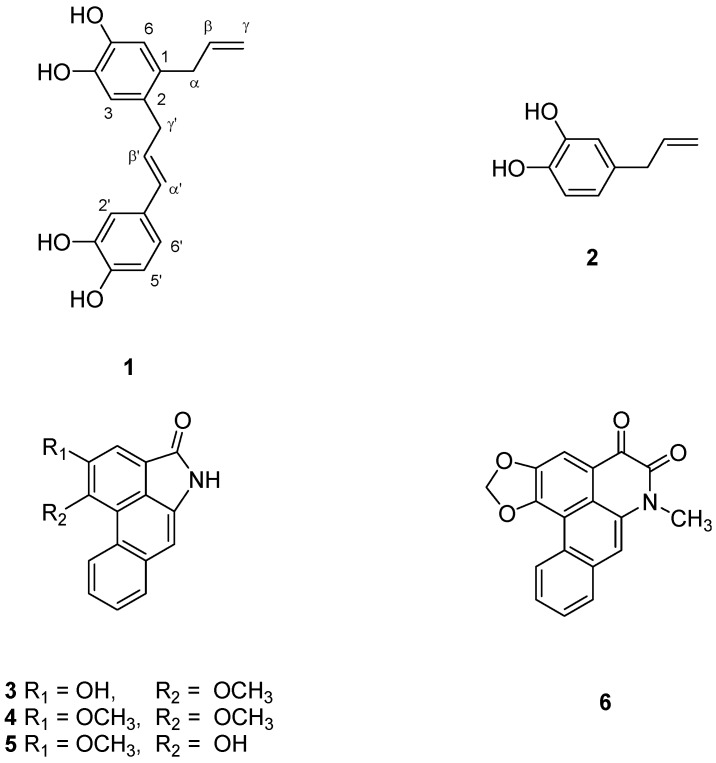
The chemical structures of compounds **1**–**6**.

*Piper betle* Linn. (Piperaceae) has been extensively used in India, China, Taiwan, Thailand and many other countries [1]. The leaves are chewed with betel nut, to improve the taste and to prevent halitosis [2,3]. Traditionally, the roots has been used for the treatment of wind-cold cough, bronchial asthma, rheumatism, stomachalgia, edema of pregnancy, and as a contraceptive [4,5]. In previous phytochemical studies, several compounds, including β-sitosteryl palmitate, 3β-acetate ursolic acid, ursolic acid, 4-allylresorcinol, stigmast-4-en-3,6-dione and aristololactam A-II, have been isolated from the roots of *P*. *betle* [6,7,8]*.* Recently, we found that the ethanolic extract of the roots of this plant exhibited anti-inflammatory effects. Chromatography of the ethanolic extract led to the isolation of a new phenolic compound, 2-(γ'-hydroxychavicol)-hydroxychavicol (**1**), together with hydroxychavciol (**2**), aristololactam A II (**3**), aristololactam B II (**4**), piperolactam A (**5**) and cepharadione A (**6**) [9,10,11,12,13] (Figure 1).

Neutrophils play a pivotal role in the defense of the human body against infections. However, activated human neutrophils are known to cause tissue damage and to play a critical role in a variety of acute and chronic inflammatory diseases [14,15]. For example, high concentrations of reactive oxygen species and elastase produced by activated neutrophils in the sputum of patients with airway mucus hypersecretion has been implicated in the pathogenesis of many pulmonary diseases including asthma, chronic obstructive pulmonary disease, cystic fibrosis and acute respiratory distress syndrome [16,17,18,19]. In a search for suitable new anti-neutrophilic inflammatory agents from natural sources, the inhibition of O_2_^•−^ production and elastase release in human neutrophil by compounds **1**–**6** were assayed. This paper describes the isolation, the determination of the structure of the new compound and the anti-inflammatory activity of the isolated compounds.

## 2. Results and Discussion

Compound **1** was obtained as a brown solid with a melting point of 73–75 °C. The EIMS gave a molecular ion at *m/z* 298 and the HREIMS spectrum gave 298.1216 (Calcd 298.1205), which corresponds to a molecular formula of C_18_H_18_O_4_. In the ^1^H-NMR spectrum of **1**, two groups of aromatic proton signals could be attributed to a set of ABX-type aromatic protons at δ_H_ 6.90 (1H, d, *J* = 2.4 Hz, H-2'), 6.74 (1H, d, *J* = 8.4 Hz, H-5'), 6.70 (1H, dd, *J* = 2.4, 8.4 Hz, H-6') and a 1,2,4,5-tetrasubstituted aromatic protons at δ_H_ 6.68 (1H, s, H-3) and 6.65 (1H, s, H-6), respectively. In addition, the signals at δ_H_ 3.27 (2H, dd, *J* = 1.2, 6.6 Hz, H-α), 5.92 (1H, m, H-β), 4.99 (1H, m, H-γ) and 4.96 (1H, dd, *J* = 2.4, 4.2 Hz, H-γ) were assigned to an allyl substituent, and another set of resonances at δ_H_ 6.24 (1H, bd, *J* = 15.6 Hz, H-α'), 6.09 (1H, td, *J* = 6.6, 15.6 Hz, H-β') and 3.34 (2H, dd, *J* = 1.2, 6.6 Hz, H-γ') were assigned to a propeneyl moiety, based on their ^1^H-^1^H COSY correlations.

**Figure 2:**
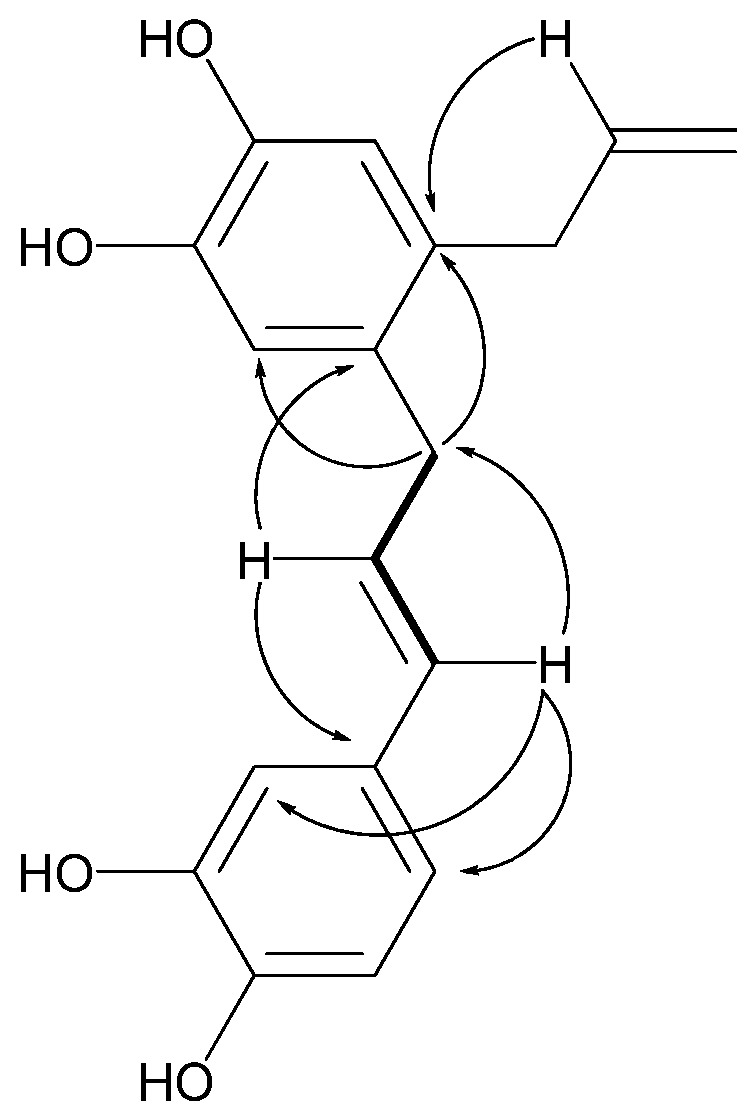
Key HMBC (arrow) and ^1^H-^1^H COSY (bold line) correlations of **1**.

In the HMBC spectrum of **1** (Table 1 and Figure 2), the methylene proton signal at δ_H_ 3.34 (H-γ') showed correlations with carbon signals at δ_C_ 117.33 (C-3) and 129.79 (C-1), which also correlated to the olefinic methane proton signal at δ_H_ 5.92 (H-β) clearly suggested that the allyl group and C-γ' were connected to C-2 and C-1 of the tetrasubstrate benzene ring, respectively. Forthemore, the olefinic methane proton signal at δ_H_ 6.09 (H-β') displayed correlations with two aromatic quaternary carbon signals at δ_C_ 130.46 (C-2) and 130.98 (C-1'), and the signals at δ_H_ 6.24 (H-α') correlated with the signals of C-2' and C-6', indicated that C-α' was located at C-1'. The coupling constant (*J*_α'-β'_ = 15.6 Hz) indicated a trans configuration between H-α and H-β. From the above data, the structure of **1** was identified as 2-(γ'-isohydroxychavicol)hydroxychavicol.

**Table 1:** ^1^H-(600 MHz) and ^13^C-NMR (150 MHz) data of compound **1** (in acetone-*d*_6_, *δ* inppm, *J* in Hz).

No.	δ_C_	δ_H_	Key HMBC (H to C)
1	129.79		
2	130.46		
3	117.33	6.68 (1H, s)	C-1, C-γ'
4	144.06		
5	144.06		
6	117.46	6.65 (1H, s)	C-2, C-5, C-α
α	37.05	3.27 (2H, dd, *J* = 1.2, 6.6 Hz)	C-2, C-6, C-γ
β	138.85	5.92 (1H, m)	C-1
γ	115.23	4.96 (1H, dd, *J* = 2.4, 4.2 Hz)	C-α, C-β
		4.99 (1H, m)	
1'	130.98		
2'	113.35	6.90 (1H, d, *J* = 2.4 Hz)	C-α', C-6', C-4'
3'	145.87		
4'	145.29		
5'	116.00	6.74 (1H, d, *J* = 8.4 Hz)	C-1', C-3'
6'	119.05	6.70 (1H, dd, *J* = 2.4, 8.4 Hz)	C-2', C-4', C-α'
α'	131.05	6.24 (1H, bd, *J* = 15.6 Hz)	C-2', C-6', C-γ'
β'	127.35	6.09 (1H, td, *J* =6.6, 15.6 Hz)	C-2, C-1'
γ'	35.98	3.34 (2H, dd, *J* = 1.2, 6.6 Hz)	C-1, C-3, C-α'

**Figure 3:**
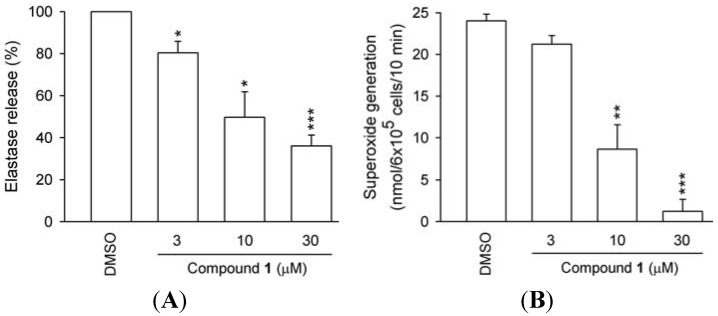
Concentration-dependent effects of compound **1** on O_2_^•−^ production and elastase release in human neutrophils. Human neutrophils were preincubated with DMSO (control) or compound **1** for 5 min before activation by FMLP/CB. (**A**) O_2_^•−^ production and (**B**) Elastase release was induced by FMLP/CB. All data are expressed as the mean ± S.E.M. (*n* = 3). * *p* < 0.025; ** *p* < 0.01; *** *p* < 0.001 compared to the control.

The *in vitro* anti-inflammatory effects of compounds **1**–**6** were tested (Table 2). Compound **2** (hydroxychavicol monomer) showed significant inhibitory effects in superoxide anion generation and elastase release (IC_50_ 0.27 and 5.78 μM; Table 2 and Figure 3).

**Table 2:** Effects of compounds on superoxide anion generation and elastase release by human neutrophils in response to FMLP/CB.

Compound	Superoxide anion		Elastase release	
	IC_50_ (μM)	Inh % ^a^	IC_50_ (μM)	Inh % ^a^
**1**	8.59 ± 2.30	94.85 ± 6.14 ***	13.14 ± 7.05	60.24 ± 3.82 ***
**2**	0.27 ± 0.09	107.12 ± 1.36 ***	5.78 ± 1.56	94.42 ± 6.49 ***
**3**	>30	4.15 ± 2.07	>30	19.36 ± 4.27 *
**4**	>30	28.96 ± 4.05 **	>30	13.65 ± 3.67 *
**5**	>30	41.06 ± 1.71 ***	>30	48.92 ± 5.32 ***
**6**	>30	43.63 ± 1.05 ***	19.19 ± 3.91	58.43 ± 2.31 ***
Sorafenib ^b^	3.01 ± 0.25		2.25 ± 0.36	

^a^ Percentage of inhibition (Inh %) at 30 μM concentration. Results are presented as the mean ± S.E.M. (*n* = 3). * *p* < 0.05; ** *p* < 0.01; *** *p* < 0.001 compared with the control value. ^b^ Sorafenib, a tyrosine kinase inhibitor, was used as a positive control.

**Figure 4:**
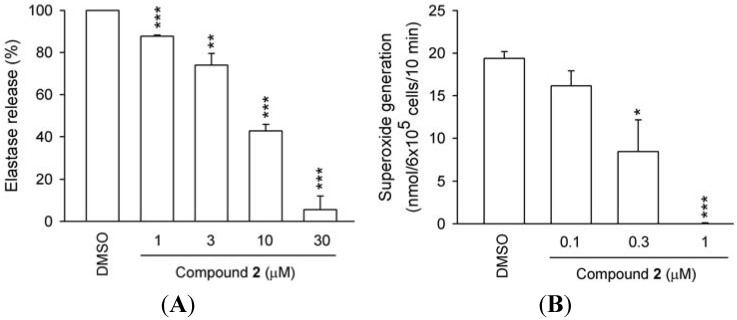
Concentration-dependent effects of compound **2** on O_2_^•−^ production and elastase release in human neutrophils. Human neutrophils were preincubated with DMSO (control) or compound **2** for 5 min before activation by FMLP/CB. (**A**) O_2_^•−^ production and (**B**) Elastase release was induced by FMLP/CB. All data are expressed as the mean ± S.E.M. (*n* = 3). * *p* < 0.025; ** *p* < 0.01; *** *p* < 0.001 compared to the control.

Compound **1** (a dimer of hydroxychavicol) also showed moderate effects in both assays (IC_50_ 8.59 and 13.14 μM; Table 2 and Figure 4). These findings suggest that compounds **1** and **2** merit further investigation as potential anti-inflammatory compounds.

## 3. Experimental

### 3.1. General

Melting points were determined using a Yanaco MP-I3 micro melting point apparatus and the thermometer was used without correction. Mass spectra were recorded using a Finnigan MAT GCQ spectrometer (EIMS). ^1^H, ^13^C, and 2D-NMR spectra were measured with a Varian VNMRS 600 MHz spectrometer.

### 3.2. Plant Material

The roots of *P. betle* Linn. were collected from Taitung County, Taiwan, in April 2011, and was identified by a taxonomist, Mr. Jun-Chih Ou*.* A voucher specimen (No.20110401) was deposited in the Department of Plant Industry, National Pingtung University of Science and Technology.

### 3.3. Extraction and Isolation

The air-dried roots of *P*. *betle* (13.6 kg) were extracted with ethanol (50 L × 2) at 50 °C for 24 h. After evaporation of the solvent *in vacuo*, the residue was partitioned between water and EtOAc to give water-soluble and EtOAc-soluble portions. The chromatography of the EtOAc soluble portion was performed using a silica gel column (70–230 mesh, 10 × 40 cm) and elution with gradient solvent of *n*-hexane−EtOAc (20:1 to 0:1) and then EtOAc−MeOH (20:1 to 1:1) to yield 16 fractions (Fr. 1 to Fr. 16). Material Fr. 7, *n*-hexane−EtOAc = 5:1 eluate, was separated over a silica gel column and eluted with *n*-hexane−EtOAc (10:1 to 1:1) and Sephadex LH-20 column with MeOH to yield hydroxychavicol (**2**, 200.3 mg). Material Fr.10, *n*-hexane−EtOAc = 2:1 eluate, was separated using Sephadex LH-20 column with MeOH to yield five subfractions (Fr. 10-1 to Fr. 10-5), of which Fr. 10-3 was repeatedly chromatographed on Sephadex LH-20 column with MeOH, silica gel column eluted with *n*-hexane−EtOAc (3:1–0:1) and preparative TLC (*n*-hexane−EtOAc = 5:4) to yield aristololactam B II (**4**, 2.4 mg), 2-(γ'-hydroxychavicol)-hydroxychavicol (**1**) and aristololactam A II (**3**, 2.1 mg). Fr. 11, *n*-hexane−EtOAc = 1:1 eluate, was re-separated on a silica gel column eluting with *n*-hexane−EtOAc (10:1–0:1) to yield piperolactam A (**5**, 3.5 mg) and cepharadione A (**6**, 4.5 mg).

*2-(γ'-Hydroxychavicol)-hydroxychavicol* (**1**). Brown solid, melting point 73–75 °C. ^1^H-NMR, ^13^C-NMR and HMBC: see Table 1. EIMS *m/z* (rel. int.) 298 [M]^+^ (6), 284 (59), 256 (23), 241 (19), 213 (39), 199 (32), 185 (100), 171 (66), 163 (28), 157 (47). HREIMS: 298.1216 (Calcd 298.1205 for C_18_H_18_O_4_).

*Hydroxychavicol* (**2**). Brown solid, melting point of 35–36 °C. ^1^H-NMR (600 MHz, acetone-*d_6_*): δ 3.21 (2H, d, *J* = 6.6 Hz, H-α), 5.04–4.95 (2H, m, H-γ), 5.93–5.87 (1H, m, H-β), 6.50 (1H, dd, *J* = 8.4, 1.8 Hz, H-6), 6.67 (1H, d, *J* = 1.8 Hz, H-2), 6.73 (1H, d, *J* = 8.4 Hz, H-5), ^13^C-NMR (150 MHz, acetone-*d_6_*) δ 40.1 (C-α), 115.2 (C-γ), 115.9 (C-5), 116.4 (C-2), 120.5 (C-6), 132.4 (C-1), 139.1 (C-β), 144.1 (C-4), 145.7 (C-3). EIMS *m/z* (rel. int.) 150 [M]^+^ (72), 131 (63), 123 (61), 103 (82), 77 (72), 51 (100).

*Aristololactam A II* (**3**). Yellow powder, melting point 270–271 °C. ^1^H-NMR (600 MHz, acetone-*d_6_*) δ 3.91 (3H, s, 4-OMe), 6.97 (1H, s, H-9), 7.44 (2H, m, H-6 and H-7), 7.51 (1H, s, H-2), 7.82 (1H, m, H-8), 9.00 (1H, m, H-5), 10.67 (1H, br s, NH). EIMS *m/z* (rel. int.) 265 [M]^+^ (68), 250 (63), 222 (60), 166 (100).

*Aristololactam B II* (**4**). Yellow powder, melting point 260–262 °C. ^1^H-NMR (600 MHz, DMSO-*d_6_*) δ 4.03 (3H, s, 4-OMe), 4.12 (3H, s, 3-OMe), 7.13 (1H, s, H-9), 7.56 (2H, m, H-6 and H-7), 7.85 (1H, s, H-2), 7.94 (1H, m, H-8), 9.11 (1H, m, H-5), 10.83 (1H, br s, NH). ^13^C-NMR (150 MHz, DMSO-*d_6_*) δ 56.9 (3-OMe), 59.9 (4-OMe), 104.7 (C-9), 109.9 (C-2), 120.0 (C-4a), 121.6 (C-1), 123.4 (C-10a), 125.5 (C-6), 126.0 (C-4b), 126.9 (C-5), 127.5 (C-7), 129.1 (C-8), 134.9 (C-8a), 135.2 (C-10), 150.5 (C-4), 154.3 (C-3), 168.5 (C=O). EIMS *m/z* (rel. int.) 279 [M]^+^ (100), 264 (24), 236 (34), 221 (23), 209 (21), 193 (35), 181 (35), 165 (49), 164 (56).

*Piperolactam A* (**5**). Yellow powder, melting point >300 °C. ^1^H-NMR (600 MHz, CD_3_OD) δ 4.09 (3H, s, 3-OMe), 6.58 (3H, s, 3-OMe), 7.15 (1H, s, H-9), 7.53 (2H, m, H-6 and H-7), 7.77 (1H, s, H-2), 7.85 (1H, m, H-8), 9.32 (1H, m,H-5). ^13^C-NMR (150 MHz, CD_3_OD) δ 57.7 (3-OMe), 107.2 (C-9), 108.9 (C-2), 116.0 (C-4a), 116.9 (C-1), 126.2 (C-10a), 126.4 (C-6), 127.7 (C-7), 128.8 (C-4b), 129.2 (C-5), 129.8 (C-8), 135.7 (C-8a), 135.9 (C-10), 149.7.5 (C-3), 151.6 (C-4), 172.3 (C=O). EIMS *m/z* (rel. int.) 265 [M]^+^ (81), 250 (52), 222 (46), 166 (100), 139 (68).

*Cepharadione A* (**6**). Orange powder, melting point >300 °C. ^1^H-NMR (600 MHz, DMSO) δ 3.74 (3H, s, NMe), 6.58 (2H, s, OCH_2_O), 7.72 (2H, m, H-6 and H-7), 7.92 (1H, s, H-9), 7.99 (1H, s, H-2), 8.11 (1H, m, H-8), 8.84 (1H, m,H-5). ^13^C-NMR (150 MHz, DMSO) δ 30.2 (NMe), 103.6 (OCH_2_O), 107.7 (C-2), 113.9 (C-4a), 114.3 (C-9), 120.4 (C-10a), 122.6 (C-1), 124.5 (C-4b), 125.9 (C-5), 127.3 (C-6), 128.2 (C-7), 128.8 (C-8), 131.6 (C-8a), 132.2 (C-10), 147.6 (C-3), 151.1 (C-4), 155.8 (11-C=O), 174.2 (12-C=O). ESIMS *m/z* (rel. int.) 328 [M+Na]^+^ (100), 320 (54), 306 [M+H]^+^ (44), 301 (15), 277 (13).

### 3.4. Anti-Inflammatory Activity

Compounds **1**–**6** were evaluated for their anti-inflammatory activity based on their inhibition of against superoxide anion generation and elastase release by human neutrophils in response to fMLP/CB. The measurements were assayed using the method described previously [19,20,21].

## 4. Conclusions

In summary, compound **1** is a new hydroxychavicol dimer and compounds **2** and **4**–**6** were isolated from the roots of *P. betle* for the first time. Hydroxychavicol monomer **2** was found to significantly inhibit superoxide anion and elastase released by human neutrophils, in response to fMLP/CB. The new compound **1** also proved to be moderately active in both anti-inflammatory assays.

## Supplementary Materials

Supplementary materials can be accessed at: http://www.mdpi.com/1420-3049/18/3/2563/s1.
