# Supplementary material for: A New Hydroxychavicol Dimer from the Roots of Piper betle
Source: Molecules. 2013 Feb 26;18(3):2563–70. doi: 10.3390/molecules18032563 (PMC6270560; doi:10.3390/molecules18032563)

Supplementary Materials

Figure 1-1. ^{1}H-NMR of compound **1**.

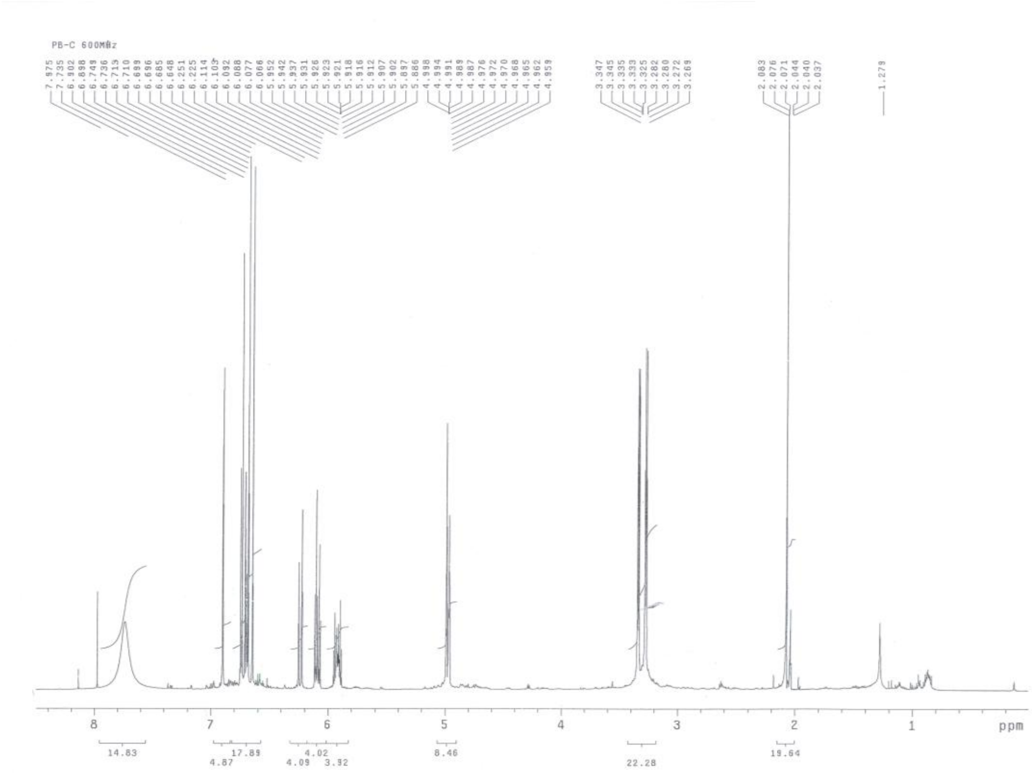

Figure 1-2. ^{13}C-NMR of compound **1**.

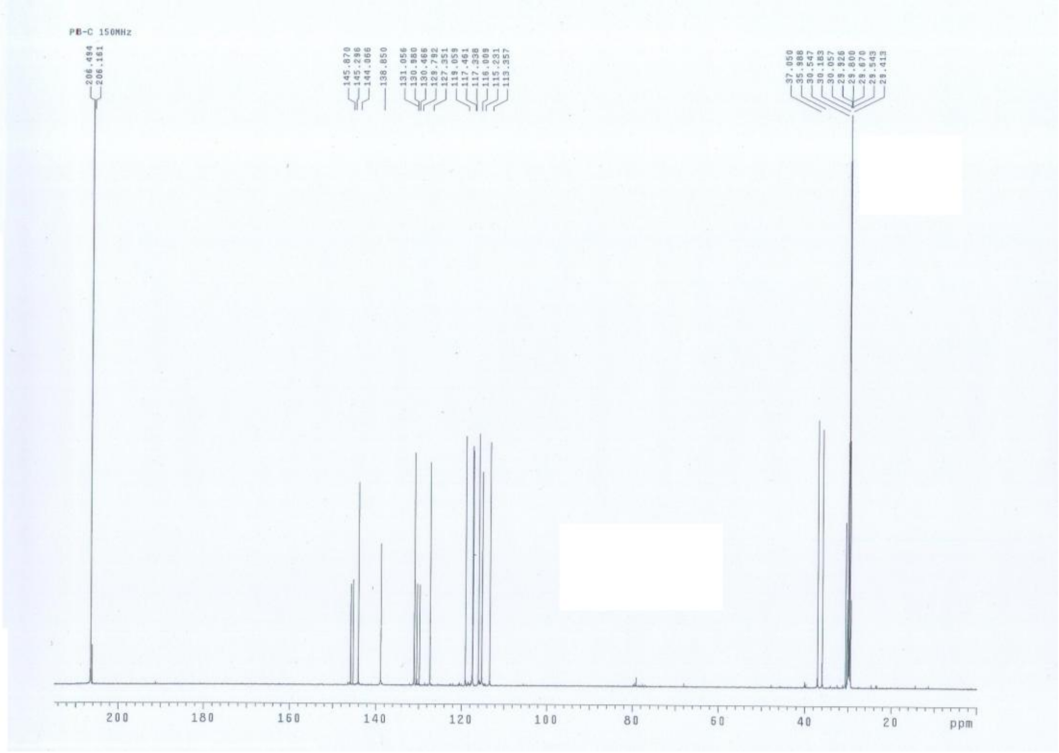

Figure 1-3. ^{1}H-^{1}H COSY of compound **1**.

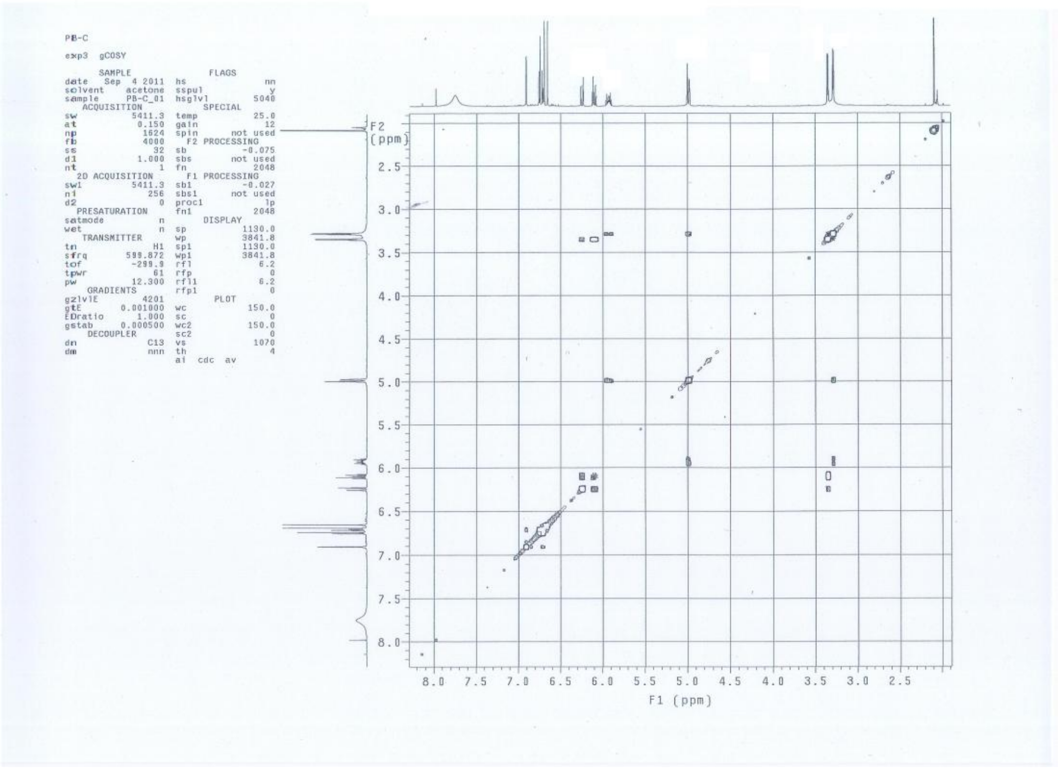

Figure 1-4. HMBC of compound **1**.

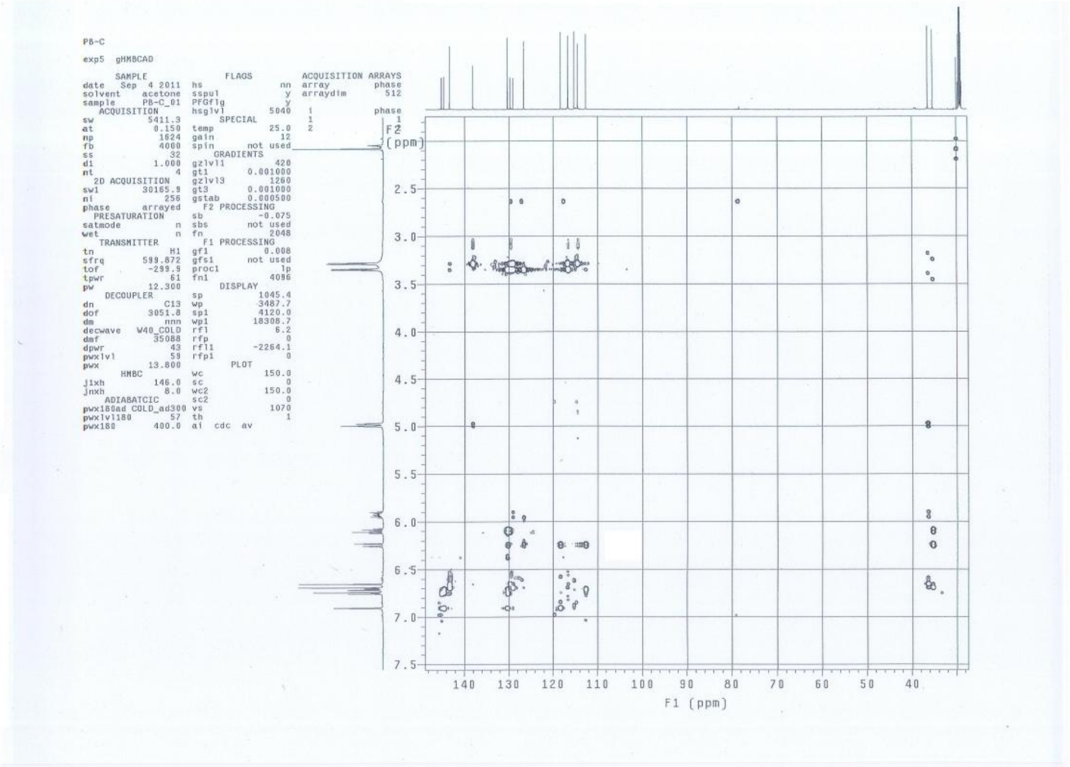

Figure 1-5. EIMS of compound **1**.

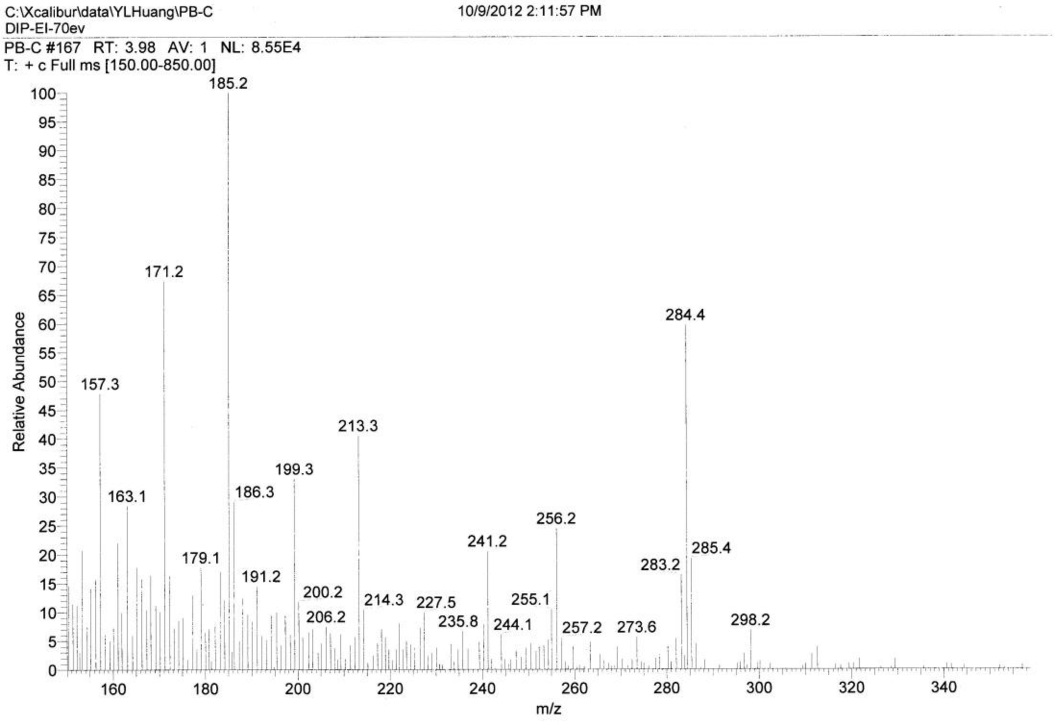

Figure 1-6. HRMS data of compound **1**.

```
LIST: hei6573_pbc-c7                    21-Nov-12 REG    : 00:31.2      #9
Samp:                                             Start  : 11:02:35   3150
Mode: EI +VE +LMR  ESCAN (EXP) UP HR NRM
Oper:                                             Inlet  :
Limt:  (   0)        .      .
    :  (553) C36.H41.O5
Peak: 1000.00 mmu    R+D: -2.0 > 60.0
Data: +/90>120   (CMASS : converted; CMASS : converted; CMASS : conver

                 93                    (mmu)
    Mass   Intensity     %RA    Flags  Delta   R+D   Composition
298.1216        2215    9.43    #       -1.0  10.0   C18.H18.O4
```

Figure 2-1. ^{1}H-NMR of compound **2**.

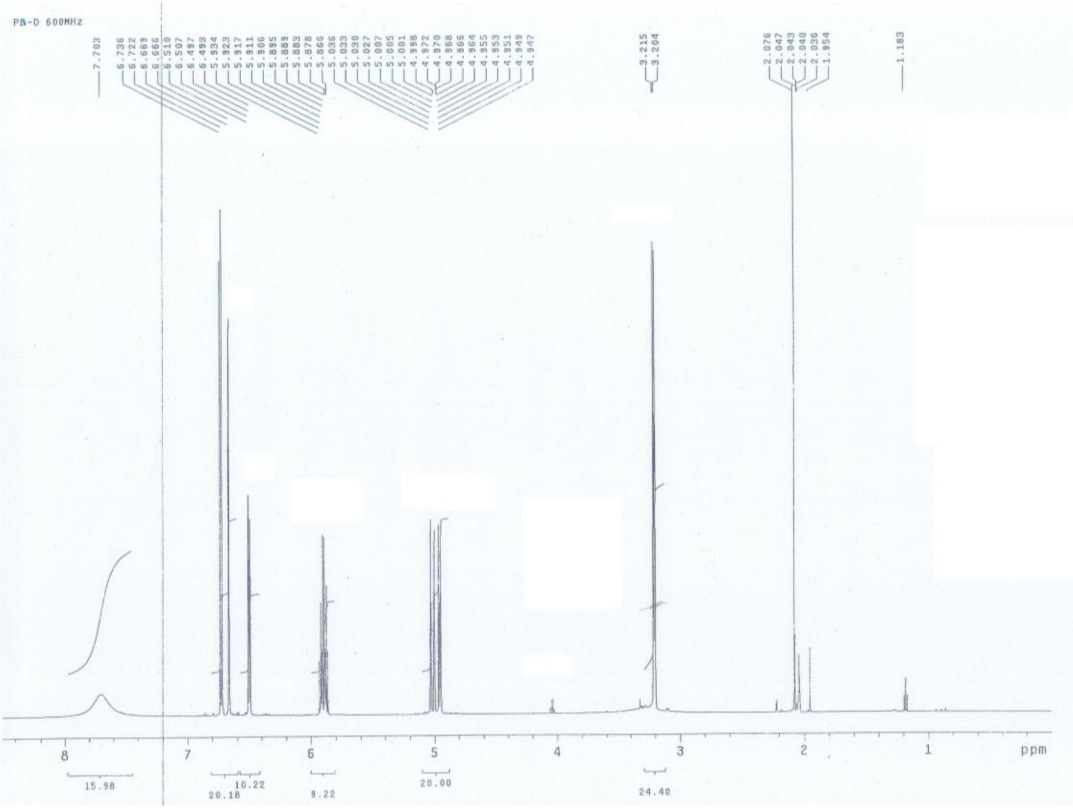

Figure 2-2. ^{13}C-NMR of compound **2**.

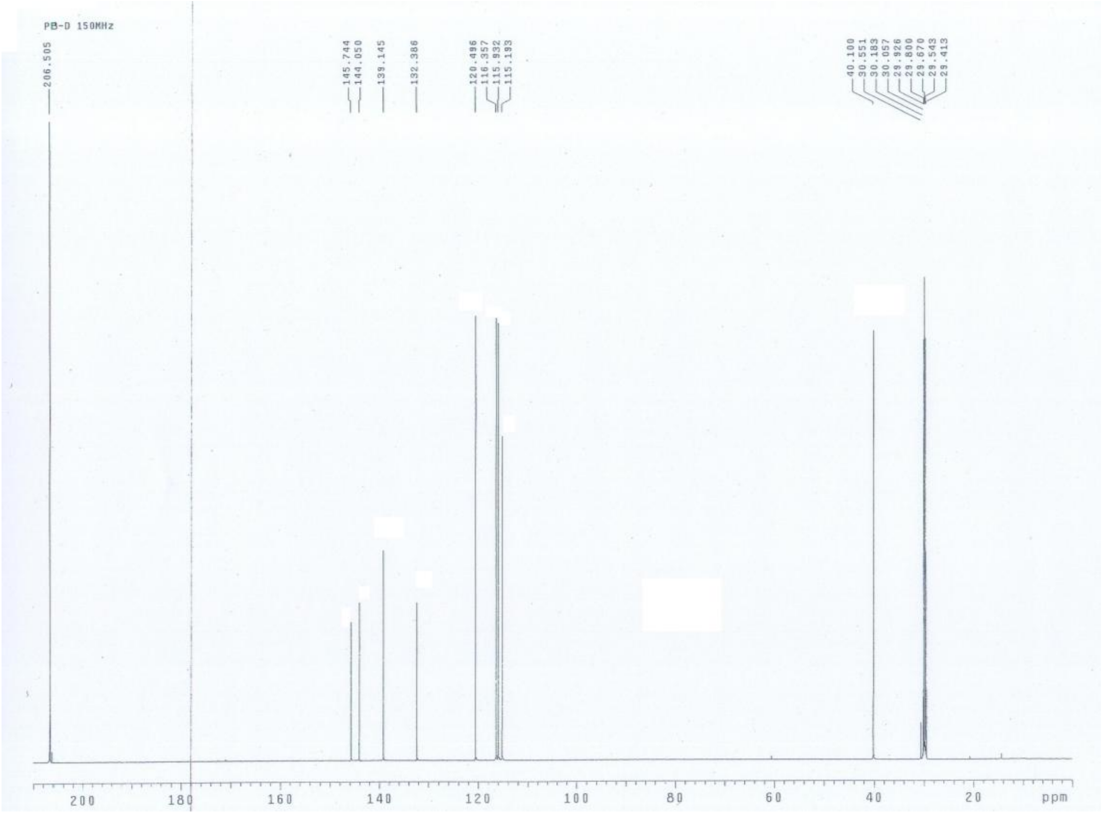

Figure 2-3. EIMS of compound **2**.

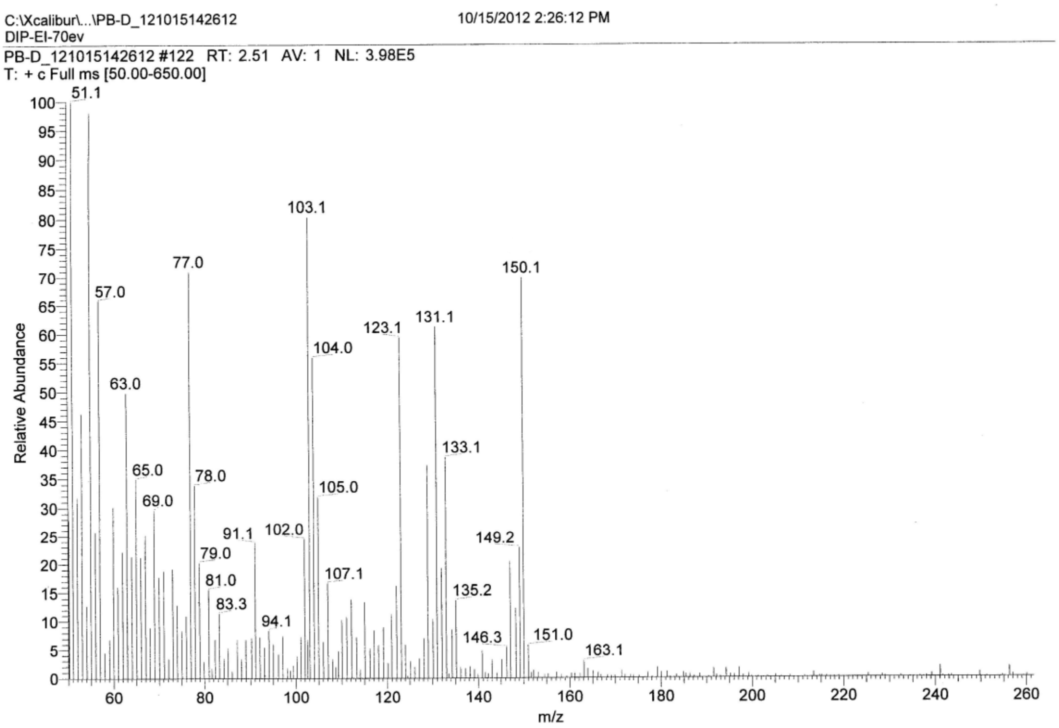

Figure 3-1. ^{1}H-NMR of compound **3**.

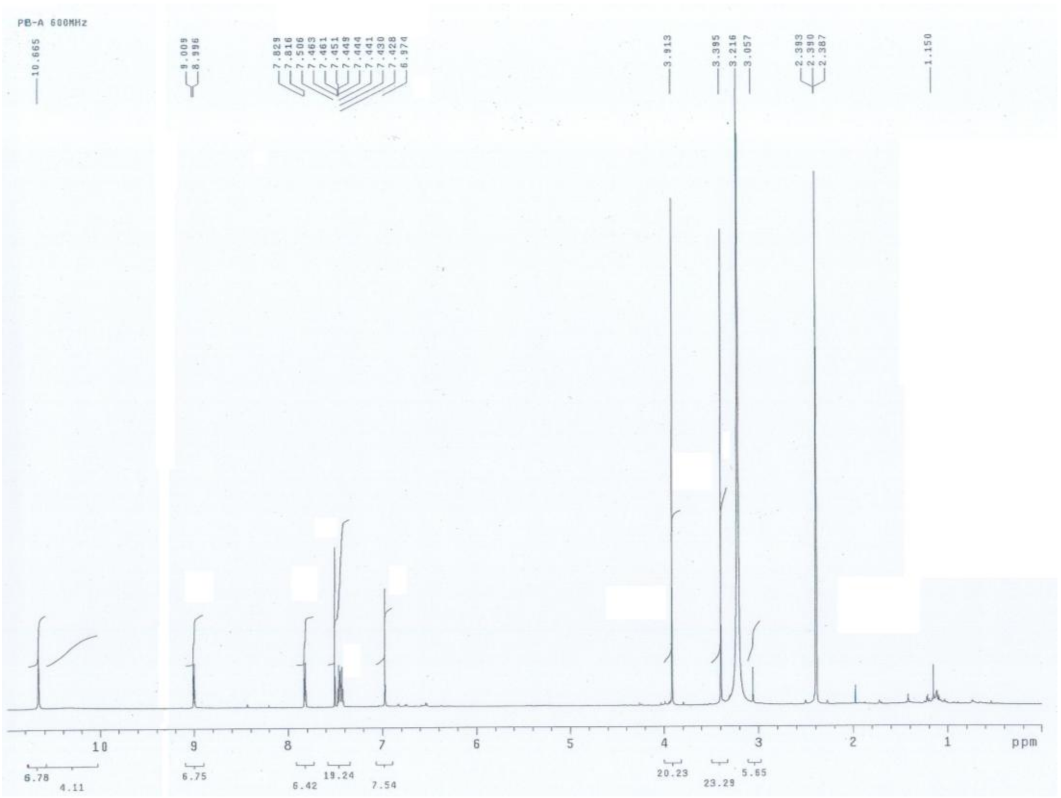

Supplement: Supplementary file 1 [file molecules-18-02563-s001.pdf]
